# Use of Baricitinib in Combination With Remdesivir and Steroid in COVID-19 Treatment: A Multicenter Retrospective Study

**DOI:** 10.7759/cureus.20620

**Published:** 2021-12-22

**Authors:** Jessica M So, Chukwuemeka Umeh, Steven Noriega, Erica Stratton, Mahendra Aseri, Rakesh C Gupta

**Affiliations:** 1 Internal Medicine, Hemet Global Medical Center, Hemet, USA; 2 Family Medicine, Hemet Global Medical Center, Hemet, USA; 3 Data Engineering and Business Intelligence, Hemet Global Medical Center, Hemet, USA; 4 Pulmonary and Critical Care Medicine, Hemet Global Medical Center, Hemet, USA

**Keywords:** hospitalized patients, mortality, cytokine storm, baricitinib, covid-19

## Abstract

Introduction

Hospitalized patients infected with severe acute respiratory syndrome coronavirus 2 (SARS-CoV-2) can develop severe complications. Baricitinib, a Janus kinase (JAK) JAK1/JAK2 inhibitor used to treat rheumatoid arthritis, has been proposed to prevent intracellular uptake of SARS-CoV-2 by targeting the angiotensin-converting enzyme 2 (ACE2) receptor, suppressing cytokine storm. We evaluated the effects of baricitinib on coronavirus disease 2019 (COVID-19) patient survival.

Methods

We conducted a retrospective study of 100 COVID-19 patients hospitalized in Southern California, United States, throughout September 2021. Univariate analysis of study variables was conducted with bivariate analysis of their relationships using chi-square and t-test with p-value <0.05 considered significant. Kaplan-Meier survival analysis was performed to compare outcomes of COVID-19 patients treated with baricitinib and those that were not.

Results

Our study included a patient population with a mean age of 62 years. Twenty-four percent of our patients were admitted to the intensive care unit (ICU), 16% were placed on mechanical ventilation, and 27% were expired. Patients receiving baricitinib were more likely to be admitted to the ICU and receive concomitant remdesivir therapy. Use of baricitinib increased median survival (p = 0.045).

Conclusion

Baricitinib administered with remdesivir and dexamethasone was shown to increase the survival of hospitalized patients with COVID-19. More studies are required to evaluate the benefits of conjunctive therapy with baricitinib, remdesivir, and dexamethasone. Though our study shows increased survival in patients receiving therapy, our study is limited by small sample size and there was not enough data to confirm whether baricitinib therapy decreased disease progression. Further studies are required.

## Introduction

Hospitalized patients infected with severe acute respiratory syndrome coronavirus 2 (SARS-CoV-2) infection can progress to severe pneumonia, acute respiratory distress syndrome, multiple organ dysfunction, septic shock, and death [1]. The cytokine release syndrome, also loosely referred to as cytokine storm, has been implicated in the sudden deterioration of disease that is caused by this highly transmissible and pathogenic coronavirus [1-4]. It is associated with the activation of immune cells and the secretion of proinflammatory cytokines such as interleukin-6 (IL-6) and tumor necrosis factor-α (TNF-α). Agents that inhibit components of this pro-inflammatory cascade have been used as treatment options to dampen the process.

Baricitinib is one of the approved agents that was given emergency use authorization by the U.S. Food and Drug Administration (FDA) to treat hospitalized patients with coronavirus disease 2019 (COVID-19) who require oxygen supplementation [1,5,6]. It is originally known to have clinical benefits for the treatment of patients with moderate-to-severe rheumatoid arthritis [7,8]. Baricitinib is an oral drug that works by intracellularly inhibiting the proinflammatory signal of several cytokines by suppressing Janus kinase (JAK) JAK1/JAK2. In terms of treating COVID-19 patients, it is thought to interrupt the passage and intracellular assembly of SARS-CoV-2 into the target cells mediated by angiotensin-converting enzyme 2 (ACE2) receptor as well as suppressing the cytokine storm [1,7].

Despite treatment advances with remdesivir and dexamethasone, reducing mortality among hospitalized COVID-19 patients remains a crucial unmet need. New treatment options are still urgently needed to reduce the high frequency of complications and deaths. There are only a few studies that evaluated the use of baricitinib in conjunction with remdesivir and dexamethasone, which showed a significant reduction in the frequency of disease progression and reduced mortality including the COV-BARRIER study [1,9,10]. Baricitinib was recently used in our two hospitals starting in September 2021 based on allocation issues. Clinically, we were observing that patients on high-flow oxygen were improving and being discharged home quicker. However, there was no data to confirm whether baricitinib was decreasing the rate of disease progression and decreasing the mortality rates in our two hospitals here in Southern California. We evaluated the effects of baricitinib among hospitalized COVID-19 patients with the standards of treatment in this retrospective cohort study.

## Materials and methods

This is a retrospective cohort study of 100 patients with COVID-19 admitted to two hospitals in Southern California, United States, in September 2021. The study population includes all patients who sought care at the two facilities for COVID-19-related symptoms and were diagnosed with COVID-19 through a positive polymerase chain reaction (PCR) nasopharyngeal swab. Patients de-identified data including patients age, sex, race, body mass index (BMI), ethnicity, marital status, comorbidities, laboratory results on admission, date of admission, date of discharge, medications they received while on admission, and disposition at discharge were extracted from the electronic medical record. The two hospitals had COVID-19 teams consisting of a pulmonary and critical care specialist and hospitalists, in consultation with an infectious disease specialist and pharmacist who decided patients’ level of care and treatment based on existing treatment guidelines. Every COVID-19 patient was seen by one of the members of the COVID-19 team.

We did a univariate analysis of study variables using means and percentages. We performed a bivariate analysis of the relationship of different study variables with baricitinib use using chi-square test and t-test, with a p-value of 0.05 considered significant. Finally, we did a Kaplan-Meier analysis to compare survival in COVID-19 patients that received baricitinib and those that did not. We did not perform a multivariate analysis to study the relationship between mortality and baricitinib use, and the effect of patients age, sex, BMI, race, ethnicity, marital status, comorbidities, the medication that patients received while in the hospital, and laboratory results on admission, because of the small sample size of our study. The calculations were performed using IBM SPSS Statistics version 27 (Armonk, NY: IBM Corp.). This study is part of the COVID-19 studies that received exempt status from the WIRB-Copernicus Group (WCG) institutional review board (IRB), and the IRB approval number of this study is 13410516.

## Results

Univariate analysis

The study included 100 patients with COVID-19. The mean age is 62 years and ranges from 23 to 94 years. Patients' mean length of hospital stay is nine days and ranges from 0 to 38 days (Table 1). The mean C-reactive protein (CRP) on admission is 9.3 and ranges from 0.13 to 18.6 and the mean body temperature on admission is 98.9°F and ranges from 96.6°F to 104.2°F. Fifty-one percent of the patients were female, 80% were whites, 24% were admitted in the intensive care unit (ICU), 16% were placed on the ventilator, and 27% died (Table 2).

**Table 1:** Univariate analysis of continuous variables.

Statistics	Mean	SD
Age (years)	61.88	16.65
Body mass index (BMI)	30.47	7.45
Length of hospital stay (days)	8.96	7.94

**Table 2:** Univariate analysis of categorical variables.

Statistics		Frequency	Percentage (%)
Gender	Female	51	51
	Male	49	49
Race	Black	10	10
	Other races	10	10
	White	80	80
Expired	No	69	72.6
	Yes	26	27.4
Ventilator use	No	84	84
	Yes	16	16
ICU	No	76	76
	Yes	24	24
Bradycardia	No	83	83
	Yes	17	17
Remdesivir	No	15	15
	Yes	85	85
Dexamethasone	No	17	17
	Yes	83	83
Baricitinib	No	73	73
	Yes	27	27

Bivariate analysis

In the bivariate analysis of continuous variables, length of hospital stay was significantly associated with baricitinib use. The mean length of hospital stay is 14 days for patients on baricitinib compared to seven days for those that did not receive baricitinib (p = 0.001). There was no difference in the age, BMI, or labs on admission in those that received baricitinib and those that did not (Table 3).

**Table 3:** Bivariate analysis of the relationship between continuous variables and use of baricitinib.

Group statistics	Baricitinib use	N	Mean	SD	p-Value
Length of hospital stay (days)	Yes	27	13.78	8.772	0.001
	No	73	7.18	6.856	
Age	Yes	27	57.96	16.068	0.154
	No	73	63.33	16.744	
Body mass index (BMI) (kg/m^2^)	Yes	27	32.73	8.08	0.065
	No	73	29.64	7.08	
C-reactive protein (CRP) (mg/dL)	Yes	27	10.6733	5.34279	0.148
	No	61	8.6938	6.07191	
Lactate dehydrogenase (LDH) (U/L)	Yes	27	458.04	264.298	0.107
	No	48	366.31	214.962	
D-dimer (ng/mL)	Yes	27	1347.04	1514.203	0.334
	No	67	1045.85	1293.399	
Ferritin (ng/mL)	Yes	22	816.286	564.9326	0.925
	No	32	800.003	653.8097	
Troponin (ng/mL)	Yes	27	0.1441	0.46033	0.883
	No	64	0.1623	0.56983	
Creatine phosphokinase (CPK) (U/L)	Yes	26	185.46	217.475	0.361
	No	40	290.85	556.218	
Platelet (10^3^/mL)	Yes	27	244.93	105.750	0.563
	No	73	262.41	142.492	
White blood cell (WBC) (10^3^/mL)	Yes	27	10.311	5.2061	0.360
	No	73	9.064	6.2936	
Creatinine (mg/dL)	Yes	12	0.8883	0.77222	0.318
	No	32	1.2850	1.26740	
Thyroid-stimulating hormone (TSH) (U/mL)	Yes	15	5.2020	16.85474	0.420
	No	22	2.2282	2.84223	
Temperature on admission (°F)	Yes	27	98.726	1.5399	0.516
	No	73	98.973	1.7287	
Systolic blood pressure on admission (mmHg)	Yes	27	128.56	23.215	0.544
	No	73	125.05	26.317	

In the bivariate analysis of categorical variables, ICU admission (p = 0.017) and remdesivir use (p = 0.011) were significantly associated with baricitinib use. Patients who received baricitinib were more likely to be in ICU and all patients that received baricitinib also received remdesivir. There is no baseline difference in those who received and who did not receive baricitinib based on gender, race, mortality, ventilator use, heart rate (HR), or dexamethasone (Table 4).

**Table 4:** Bivariate analysis of the relationship between categorical variables and use of baricitinib. HR: heart rate; ICU: intensive care unit

Variable		Use of baricitinib		p-Value
		No	Yes	
Gender	Male	34 (69.4%)	15 (30.6%)	0.425
	Female	39 (76.5%)	12 (23.5%)	
Race	Black	8 (80%)	2 (20%)	0.569
	Other races	6 (60%)	4 (40%)	
	Whites	59 (73.7%)	21 (26.3%)	
Expired	No	53 (76.8)	16 (23.2%)	0.137
	Yes	16 (61.5%)	10 (38.5%	
Ventilator	No	64 (76.2%)	20 (23.8%)	0.1
	Yes	9 (56.2%)	7 (43.8%)	
ICU	No	60 (78.9%)	16 (21.1%)	0.017
	Yes	13 (54.2%)	11 (45.8%)	
Bradycardia (HR <50)	No	63 (75.9%)	20 (24.1%)	0.148
	Yes	10 (58.8%)	7 (41.2%)	
Tachycardia (HR >100)	No	37 (82.2%)	8 (17.8%)	0.06
	Yes	36 (65.5%)	19 (34.5%)	
Remdesivir use	No	15 (100%)	0 (0%)	0.011
	Yes	58 (68.2%)	27 (31.8%)	
Dexamethasone use	No	12 (70.6%)	5 (29.4%)	0.806
	Yes	61 (73.5%)	22 (26.5%)	

Kaplan-Meier analysis

**Figure 1:**
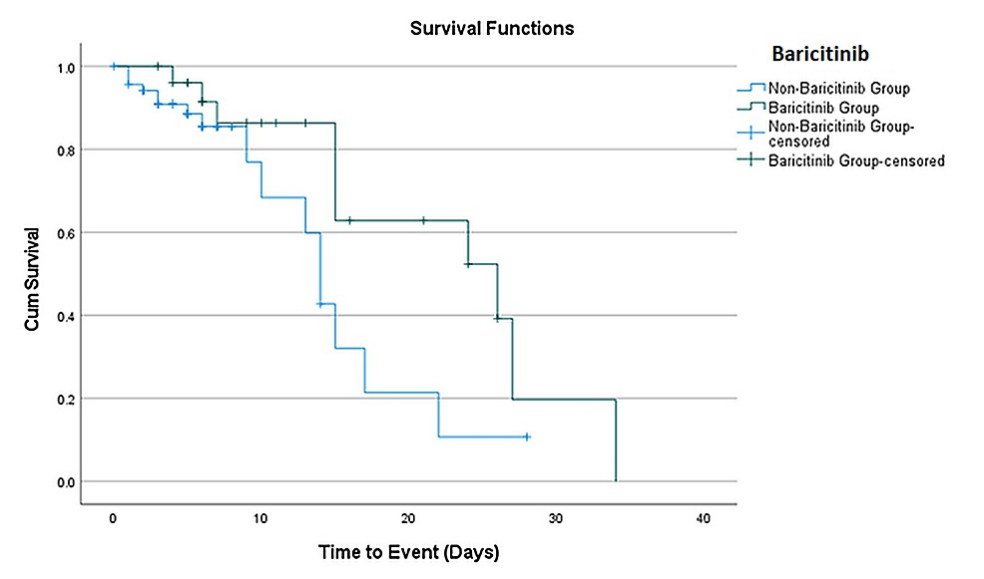
Kaplan-Meier survival curve of baricitinib use.

Kaplan-Meier analysis shows that patients that received baricitinib have increased survival compared to those that did not receive it. The median survival for those who received baricitinib is 26 days (95% CI: 11.7-40.3) while it is 14 days (95% CI: 12.4-15.6) for those who did not receive baricitinib (p = 0.045) (Figure 1).

Side effects reported with baricitinib

Some side effects were reported in patients that received baricitinib. Thirty-three percent of the patients had a minor elevation of liver enzymes after baricitinib was started, which was not severe enough to stop the medication. The dose-adjusted baricitinib (1 mg daily) was stopped in only one patient (3.7%) with underlying severe acute kidney injury who developed elevated liver enzymes after baricitinib was started. However, the liver enzymes returned to normal after baricitinib was stopped. Eleven percent of the patients who received baricitinib had mild creatinine elevation, 15% had transient bradycardia, and none had a fever (Table 5). Most patients (74%) received 4 mg of baricitinib, while 26% had their dose adjusted based on their renal function. 

**Table 5:** Side effects reported with baricitinib use.

Side effects reported	Frequency	Percentage (%) (N = 27)
Elevated liver enzymes	9	33.3
Elevated creatinine	3	11.1
Fever	0	0
Bradycardia	4	14.8
Treatment stopped due to side effect	1	3.7

## Discussion

This retrospective cohort study showed that patients who received baricitinib had increased survival compared to those who did not receive the drug. The median survival for those that received baricitinib was 26 days, while it was 14 days for those that did not receive baricitinib. We considered that this was a short period to observe this small sample size of 100 patients with COVID-19, along with the fact that only 27% of these patients were treated with baricitinib. However, this survival rate benefit using this agent in conjunction with the standard of treatment with remdesivir and dexamethasone was encouraging. The patients on baricitinib in our study received an average of six days of baricitinib prior to discharge or death. Per FDA guidelines, the dosing strategy for baricitinib is 4 mg daily for 14 days or until hospital discharge or death [6]. Additionally, a total of 27 patients who received baricitinib were also treated with remdesivir, but only 25 of those 27 patients received dexamethasone. We did not pursue a multivariate proportional hazard analysis due to our small sample size. 

Limited studies are showing the benefits of combination therapy with baricitinib, remdesivir, and dexamethasone. In the COV-BARRIER study, treatment with baricitinib reduced 28-day all-cause mortality by 38.2% compared with placebo [1]. The study by Izumo et al. evaluating the clinical impact of the combination therapy showed that the 28-day mortality rate was low at 2.3% (1/44 patients) and had a median hospitalization duration of 11 days and time to recovery of nine days [9]. In the ACTT-2 study, baricitinib plus remdesivir was superior to remdesivir alone in reducing recovery time and accelerating improvement in clinical status among patients with COVID-19 [11]. They showed that the 28-day mortality was 5.1% in the combination group and 7.8% in the control group [11]. Overall, these studies showed a mortality benefit with each of these agents or in combination with each other. However, we do need more randomized studies evaluating the effects of this combination therapy.

We also observed that the mean length of hospital stay was 14 days for patients on baricitinib compared to seven days for those that did not receive baricitinib (p = 0.001). This could be attributed to starting baricitinib later during hospitalization in patients who did not improve on remdesivir and steroid therapy. It could also be explained by the fact that patients who received baricitinib were much sicker, possibly in sepsis, because those who received baricitinib in our study were more likely to be in the ICU. We observed that 45.8% of the patients who received baricitinib were more likely to be in the ICU. On an average, the ICU patients stayed longer in the hospital than non-ICU patients. At this time, we cannot differentiate between those patients who received baricitinib earlier in the course compared to those who received it later. The study by Izumo et al. showed that the median duration of ICU stay was as short as six days among patients receiving baricitinib therapy [9]. As they commented, the length of ICU stay is a critical problem in patients with severe COVID-19. We will need to do further studies to differentiate why certain patients were excluded from treatment with baricitinib.

Around 74% of the patients in our study received 4 mg of baricitinib, while 26% had their dose adjusted based on their renal function. We also observed that 33% of the patients had a minor elevation of liver enzymes after baricitinib was started, which was not severe enough to stop the medication. Additionally, the dose-adjusted baricitinib (1 mg daily) was stopped in only one patient (3.7%) with underlying severe acute kidney injury who developed elevated liver enzymes after baricitinib was started. However, the liver enzymes returned to normal after baricitinib was stopped. The FDA guidelines recommended interrupting baricitinib if increases in the liver enzymes (alanine transaminase {ALT} and aspartate transaminase {AST}) were seen. However, it did not indicate at what elevated levels to hold the medication so that drug-induced liver injury could be evaluated. In large clinical trials in rheumatoid arthritis, serum liver enzyme values were elevated in 17% of baricitinib-treated patients compared to 11% in placebo patients. These elevations were more than three times the upper limit of normal in only 1-2% of patients on baricitinib; however, they were typically mild and transient [12,13]. Those studies did not report any clinically apparent liver injury associated with baricitinib [12]. Additionally, baricitinib has the potential to reactivate hepatitis B so we evaluated the status of the 27 patients in our study that received baricitinib. It was observed that 63% of our patients tested negative while the lab test was not done in 33.3% or was inconclusive in 3.7% of our patients.

Regarding adverse events, studies including the ACTT-2 study and COV-BARRIER study reviewed emergent adverse events, serious adverse events, infections, and venous thromboembolic events. They noted that the frequencies were similar between the baricitinib and placebo groups, and no new safety signals were detected [1,11]. We observed in our study that 11% of the patients who received baricitinib had mild creatinine elevation, 15% had transient bradycardia, and none had a fever. Though our finding is consistent with previous randomized controlled studies that showed no serious adverse effects with baricitinib, more observational studies with a large sample size are needed to report the side effects of baricitinib in clinical practice as the medication becomes more widely used in the COVID-19 treatment.

Limitations of the study

The present study had several limitations. Firstly, the study was conducted at only two centers in Southern California. Secondly, only a few patients that received baricitinib were included in the study (N = 27), which limited our multivariate analysis ability. Lastly, our study is a retrospective observational study, and there could have been some unmeasured or unknown confounders that have influenced the outcome of our study. Despite these limitations, this study shows the positive outcomes in patients who received a combination of baricitinib, remdesivir, and dexamethasone and highlights the possible areas of future research.

## Conclusions

In summary, our results show that patients that received baricitinib together with remdesivir and dexamethasone had increased survival compared to those who did not receive the drug. However, our study is limited by small sample size. Therefore, there is a need for more extensive studies to evaluate the effects of baricitinib when combined with remdesivir and dexamethasone. Furthermore, for our patients, some of them were started on baricitinib after they failed to improve on remdesivir and dexamethasone. Thus, there is a need to investigate the difference in the effect of baricitinib when started concurrently with remdesivir and dexamethasone and when started only in patients who did not improve on remdesivir and dexamethasone.
